# Diagnosis and Management of Patent Foramen Ovale for Stroke Prevention: An Australian and New Zealand Consensus Statement Developed by a Modified Nominal Group Approach

**DOI:** 10.5694/mja2.70199

**Published:** 2026-05-13

**Authors:** Brian R. Chambers, Lauren M. Sanders, Amanda Gilligan, Carlos Garcia‐Esperon, Jan Ho, John Fink, Matias Yudi, Matthew Lee‐Archer, Vimal Stanislaus, Andrew A. Wong

**Affiliations:** ^1^ Austin Health Melbourne Victoria Australia; ^2^ University of Melbourne Melbourne Victoria Australia; ^3^ St Vincent's Hospital Melbourne Melbourne Victoria Australia; ^4^ Epworth Healthcare Melbourne Victoria Australia; ^5^ John Hunter Hospital Newcastle New South Wales Australia; ^6^ Hunter Medical Research Institute, University of Newcastle Newcastle New South Wales Australia; ^7^ Stroke Research Centre, Perron Institute for Neurological and Translational Science Perth Western Australia Australia; ^8^ Sir Charles Gairdner Hospital Perth Western Australia Australia; ^9^ Christchurch Hospital Christchurch Canterbury New Zealand; ^10^ Launceston General Hospital Launceston Tasmania Australia; ^11^ University of Tasmania Hobart Tasmania Australia; ^12^ Alfred Health Melbourne Victoria Australia; ^13^ Monash University Melbourne Victoria Australia; ^14^ Royal Brisbane and Women's Hospital Brisbane Queensland Australia; ^15^ University of Queensland Brisbane Queensland Australia

**Keywords:** cardiac imaging techniques, cardiovascular abnormalities, Doppler, neuroimaging, risk assessment, risk factors, stroke, ultrasonography

## Abstract

**Introduction:**

Patent foramen ovale (PFO) is implicated in 25%–50% of cryptogenic strokes in patients aged < 60 years. Recent clinical trials demonstrated the benefit of PFO closure in selected patients. However, there is considerable variability in Australian and New Zealand clinical practice regarding investigation and management approaches. A multidisciplinary consensus group comprising stroke neurologists and an interventional cardiologist from major centres employed a modified nominal group technique to develop evidence‐based recommendations for standardising PFO‐associated stroke management.

**Main Recommendations:**

Twelve recommendations were developed across three domains.

- For patient selection: Universal PFO screening for cryptogenic stroke patients aged ≤ 60 years, with selective screening for patients aged > 60 years with embolic stroke of undetermined source, absent vascular risk factors and excluded atrial fibrillation.
- For diagnostic investigations: Transcranial Doppler (TCD) bubble study as preferred first‐line screening where available, with transthoracic echocardiography as an alternative when TCD is unavailable and transoesophageal echocardiography for confirmation before closure consideration.
- For treatment decisions: Incorporation of the PFO‐Associated Stroke Causal Likelihood (PASCAL) classification system rather than the Risk of Paradoxical Embolism (RoPE) score alone, consideration of TCD grading results for risk stratification and mandatory multidisciplinary heart–brain team evaluation for all closure decisions.

**Changes in Management as a Result of This Consensus Statement:**

These recommendations will standardise practice through enhanced TCD service provision, structured heart–brain team development and evidence‐based patient selection using the PASCAL classification. The emphasis on TCD as first‐line screening represents a departure from traditional transthoracic echocardiography‐based approaches. Implementation will improve patient outcomes through appropriate intervention in suitable candidates while avoiding unnecessary procedures in those unlikely to benefit, thereby promoting equitable access to optimal PFO management across Australia and New Zealand.

## 1 Introduction

Patent foramen ovale (PFO)—a normal embryological communication between the cardiac atria—persists into adulthood in about 25% of the population [1, 2]. PFO has progressively emerged as being causally involved in 10% of all strokes in patients aged 18–60 years [2], and accounts for 30% and 10% of ischaemic strokes in people aged ≤ 50 and > 50 years, respectively [3]. The potential mechanisms of ischaemic stroke in people with PFO include paradoxical embolism from venous thrombosis, thrombus formation within the PFO tunnel and associated atrial arrhythmias [1, 4]. PFO also remains an important aetiology to consider in cases of cryptogenic stroke, responsible for 43.9% and 28.3% of cryptogenic strokes (defined as strokes of undetermined mechanism following routine first‐pass investigation [5]) in patients aged ≤ 55 and > 55 years, respectively [6].

Current international guidelines recommend PFO closure for patients aged 18–60 years with PFO‐associated stroke and high‐risk features [1, 7, 8, 9], utilising the PFO‐Associated Stroke Causal Likelihood (PASCAL) classification, which combines the Risk of Paradoxical Embolism (RoPE) score with anatomical PFO characteristics [10]. Despite this evidence, there is variation within Australian and New Zealand clinical practice regarding PFO investigation methods, patient selection criteria and treatment decisions.

The complexity of PFO management requires multidisciplinary collaboration between stroke neurologists and cardiologists. Yet, many Australian and New Zealand centres lack structured approaches to this decision‐making process. Furthermore, access to optimal diagnostic modalities, particularly the transcranial Doppler (TCD) bubble test, remains limited across many regions.

To address these challenges and promote standardised, evidence‐based care, a multidisciplinary group of stroke specialists and an interventional cardiologist convened to develop consensus recommendations for the management of PFO‐associated stroke. This initiative aimed to provide practical guidance for clinicians while advocating for improved service provision and standardised care pathways.

## 2 Methods

A structured consensus development process was employed using a modified nominal group technique. The consensus development methodology followed principles outlined in the ACCORD (Table S1) guidelines for consensus‐based research [11]; the study was not registered. All consensus activities were conducted in English. No translation services or plain language summaries were required.

A multidisciplinary expert panel was assembled, comprising nine stroke neurologists and one interventional cardiologist from major tertiary centres, with representation from six Australian and New Zealand regions (Christchurch, New South Wales, Queensland, Tasmania, Victoria and Western Australia). BC selected the panel members based on their clinical expertise, research contributions and active involvement in multidisciplinary stroke care.

A comprehensive literature review, focusing on recent clinical trials, meta‐analyses and international guidelines for PFO management, was conducted before the consensus meetings and the publications were shared with all panellists for review. Based on clinical experience and current evidence, initial recommendations were drafted and grouped into three domains: patient selection for PFO testing, diagnostic investigation modalities and treatment selection and management.

Two formal consensus meetings were conducted via videoconference in July 2025. The meetings followed a structured format, including background evidence presentation, systematic review of each draft statement, open discussion allowing all participants to contribute perspectives, iterative refinement of wording based on group feedback and identification of areas requiring further modification. Between formal meetings, a smaller working group refined the wording of the recommendations based on discussions, ensuring consistency with evidence and clinical practice while addressing the concerns raised during group sessions.

Final recommendations were subject to anonymous electronic voting to minimise groupthink and allow honest assessment of each statement. Each panel member voted to ‘agree’, ‘disagree’ or ‘abstain’ for each statement. Participants who were unable to attend specific voting sessions were provided with finalised statement wording and allowed to vote independently, thus ensuring comprehensive participation in the consensus process. All recommendations met consensus, defined as majority agreement among participating experts (> 75%), and have been included in this statement.

## 3 Recommendations

The consensus process yielded 12 recommendations across three domains that reflect current expert opinion and best available evidence (Table 1). These recommendations align closely with recent European Stroke Organisation guidelines [1] while providing Australian and New Zealand‐specific implementation guidance. The emphasis on TCD screening and structured multidisciplinary decision‐making reflects evolving international best practices and local service‐delivery considerations. The full list of recommendations is included in Table S2, which also provides the level of consensus for each recommendation and classifies them using the GRADE framework [12]. The Australasian Stroke Academy (https://strokeacademy.com.au/) has endorsed these recommendations. Clinicians are encouraged to exercise judgement and adapt these recommendations to individual circumstances.

**TABLE 1:** Summary of consensus recommendations.

Description	Grade^a^
Patient selection	
Recommendation 1: Patients aged ≤ 60 years with cryptogenic stroke should undergo screening for PFO	A1
Recommendation 2: Patients aged > 60 years with embolic stroke of undetermined source, absence of vascular risk factors and no atrial fibrillation detected on prolonged monitoring may be considered for PFO screening	B1
Diagnostic investigations	
Recommendation 3: The historical gold standard for PFO detection is TOE, but this is not the preferred screening investigation for PFO detection	A1
Recommendation 4: A TCD bubble study should be considered as a first‐line screening investigation for PFO detection	B1
Recommendation 5: Where TCD is not available, TTE with bubble study is an alternative screening investigation, although it has lower sensitivity for PFO detection	B1
Recommendation 6: In patients with a positive TCD bubble study under consideration for percutaneous PFO closure, the presence of PFO should be confirmed by TOE. TOE will also reveal high‐risk features for stroke recurrence, including large PFO and associated atrial septal aneurysm	A1
Treatment decisions	
Recommendation 7: A high RoPE score alone does not predict benefit from PFO closure and should therefore not be the principal basis of treatment decision‐making.	B1
Recommendation 8: The PASCAL classification system combines the RoPE score and high‐risk PFO features and should be incorporated into decision‐making for PFO closure	B1
Recommendation 9: Spontaneous right‐to‐left shunt and high‐grade right‐to‐left shunt with the Valsalva manoeuvre detected by TCD bubble study are predictors of increased stroke recurrence risk and should be incorporated into treatment decision‐making	B1
Recommendation 10: Patients with PFO‐associated stroke and high‐risk features for stroke recurrence should be considered for PFO closure.	B1
Recommendation 11: PFO closure decisions should be made by a multidisciplinary heart–brain team that includes a stroke physician and a structural heart cardiologist	GPP
Recommendation 12: In patients with PFO‐associated stroke, other factors, including concurrent deep vein thrombosis and pulmonary embolism, and demonstration of thrombophilia, should also be considered in treatment decision‐making	GPP

Abbreviations: GPP, good practice point; GRADE, Grading of Recommendations Assessment, Development, and Evaluation; PASCAL, Patent Foramen Ovale‐Associated Stroke Causal Likelihood; PFO, patent foramen ovale; RoPE, Risk of Paradoxical Embolism; TCD, transcranial Doppler; TOE, transoesophageal echocardiography; TTE, transthoracic echocardiography.

^a^ The quality of the supporting evidence was defined as high (A), moderate (B) or low (C) and the strength of the recommendations was classified as strong (1) or conditional (2) (Table S2).

## 4 Patient Selection for PFO Testing

### 4.1 PFO Screening in Patients Aged ≤ 60 Years

Recent randomised controlled trials have demonstrated the efficacy of percutaneous PFO closure in preventing recurrent stroke in carefully selected patients [13, 14, 15, 16, 17, 18, 19]. A meta‐analysis of six major trials showed that in patients aged 18–60 years, there was a 60% relative risk reduction in recurrent ischaemic stroke with PFO closure compared with medical therapy alone [20]. This translates to an absolute risk reduction of 1.7% at 2 years [20]. The benefits varied with patient characteristics and PFO features, underscoring the importance of appropriate patient selection [20, 21, 22].

The consensus strongly supports universal PFO screening in patients aged ≤ 60 years with cryptogenic stroke, reflecting high PFO prevalence in this population and established treatment benefits. This approach aligns with international guidelines [1, 7, 8, 9] and acknowledges that determining stroke mechanisms can be challenging because apparent small‐vessel infarcts may sometimes represent embolic events. The use of ‘cryptogenic stroke’ rather than the more restrictive ‘embolic stroke of undetermined source’ definition reflects clinical reality, where traditional lacunar‐appearing strokes in young patients may be embolic [23] (*Recommendation 1*).

### 4.2 PFO Screening in Patients Aged > 60 Years

Although long‐term randomised controlled trials are needed, observational studies have demonstrated the safety and preliminary efficacy of PFO closure in reducing the risk of recurrent stroke in patients aged > 60 years [24, 25, 26]. The consensus panel acknowledged the European Stroke Organisation's 2024 position that ‘no evidence‐based recommendation can reasonably be provided’ for patients aged > 60 years [1], but agreed that individual assessment in carefully selected cases may be warranted, particularly in the early 60s age group, where the 60‐year threshold represents an arbitrary boundary. The European Society of Cardiology recommends PFO closure in patients aged 18–65 years [2]. However, a small post hoc subgroup analysis of DEFENCE‐PFO randomised controlled trial data indicates that PFO closure benefits may be greater in patients aged ≥ 60 years (*n* = 34) than in those aged < 60 years (*n* = 86) [27].

Thus, a relatively selective approach was endorsed for this age cohort, focusing on individuals with embolic stroke of undetermined source characteristics and minimal vascular risk factors and excluding those with atrial fibrillation. This reflects the decreased likelihood of PFO causality with advancing age and competing stroke mechanisms, while recognising that some older patients may still benefit from evaluation (*Recommendation 2*).

### 4.3 Diagnostic Investigations

Table 2 compares the ultrasound modalities available for detecting PFO in Australia and New Zealand. Historically, transoesophageal echocardiography (TOE) has been the gold standard for PFO detection [28], followed by transthoracic echocardiography (TTE). However, TOE is a semi‐invasive, inconvenient and stressful procedure for patients [28]. Furthermore, some PFOs may still be missed or misdiagnosed with TOE (sensitivity, 89.2% [95% confidence interval (CI), 81.1%–94.7%]; specificity, 91.4% [95% CI, 82.3%–96.8%]) [25] and TTE (sensitivity, 45.1% [95% CI, 30.8%–60.3%]; specificity, 99.6% [95% CI, 96.5%–99.9%]) [29]. Moreover, these procedures may be performed without a bubble study, necessitating a follow‐up test to perform the bubble study. TOE is also typically done on sedated patients, and both TOE and TTE are usually done with the patient lying on their left side, so the Valsalva manoeuvre may be performed suboptimally. In addition, there may be a lack of timely access to TOE and TTE in public hospitals [30].

**TABLE 2:** Comparison of ultrasound modalities available for detecting patent foramen ovale (PFO) in Australia and New Zealand.

Feature/modality	TCD bubble study	TTE (including bubble study)	TOE (including bubble study)
Availability^a^	Readily available in a few public hospitals; not available in private practice	Long wait time in most public hospitals; readily available in most private cardiology practices	Long wait time in most public hospitals; readily available in some private cardiology practices
Interpretation^a^	Detects right‐to‐left shunt (e.g., PFO or pulmonary AVM)	Specifically tests for PFO	Specifically tests for PFO
Accuracy^a^	+++ Probably superior to TTE and TOE	++	++ Considered to be the gold standard, but sensitivity is affected by poor Valsalva manoeuvre in sedated patients and left lateral positioning
Sensitivity	96.1% (95% CI, 93.0%–97.8%) [31]	45.1% (95% CI, 30.8%–60.3%) [31]	89.2% (95% CI, 81.1%–94.7%) [29]
Specificity	92.4% (95% CI, 85.5%–96.1%) [31]	99.6% (95% CI, 96.5%–99.9%) [31]	91.4% (95% CI, 82.3%–96.8%) [29]
Additional information^a^	Results graded from 0 to 5		Detects high‐risk features for stroke recurrence (e.g., large anatomical defect, hypermobile atrial septum, ASA)
Other comments^a^		Usually performed without a bubble study unless specifically requested	High‐risk features are often not reported

Abbreviations: ASA, atrial septal aneurysm; AVM, arteriovenous malformation; CI, confidence interval; TCD, transcranial Doppler; TOE, transoesophageal echocardiography; TTE, transthoracic echocardiography.

^a^ Based on clinical experience, where + represents lowest and +++ represents highest accuracy.

By contrast, pooled sensitivity and specificity of TCD are 96.1% (95% CI, 93.0%–97.8%) and 92.4% (95% CI, 85.5%–96.1%), respectively [31]. In addition to its superior sensitivity for PFO detection (compared with TOE and TTE), it is non‐invasive, cost‐effective, highly feasible and can be performed with optimal Valsalva manoeuvres [28, 31, 32, 33]. TCD can also be used to screen for pulmonary arteriovenous malformations, and computed tomography pulmonary angiography can be performed when PFO is not confirmed in the presence of TCD‐detected right–left shunting [34, 35]. Therefore, the consensus recommends the TCD bubble study as the preferred first‐line screening modality for PFO.

The recommendation for TCD as a first‐line screening tool has implications for service delivery in Australia and New Zealand. Many centres currently lack TCD capability, limiting access to optimal PFO detection. The consensus strongly advocates for enhanced TCD service provision, with participants noting that the lack of TCD services in some regions represents a considerable gap in stroke care.

Rather than a screening tool, TOE is recognised as a tool for confirming the presence of a PFO and identifying high‐risk anatomical features before considering closure. This staged approach optimises resource utilisation while ensuring appropriate anatomical characterisation for treatment decisions. The utility of TOE in assessing for other cardiac sources of embolism and in defining precise anatomical features remains crucial for pre‐procedural planning (*Recommendations 3–6*).

### 4.4 Treatment Decisions

Tables 3 and 4 show an overview of the RoPE score and PASCAL classification systems. The RoPE score is the sum of points for clinical characteristics and points for the age category, with a maximum score of 10 [10, 20]. Although useful for estimating the probability of PFO presence and its culpability in the index stroke [36, 37], the RoPE score does not provide comprehensive information for patient selection because it does not permit the inclusion of high‐risk PFO functional and structural features, such as shunt size, atrial septal aneurysm with a > 10 mm septum hypermobility, large PFO (> 2–3 mm of separation between ostium primum and ostium secundum), long tunnel (> 10 mm) and the presence of a right atrial Chiari network or prominent Eustachian valve [20, 38, 39] (*Recommendation 7*).

**TABLE 3:** The Risk of Paradoxical Embolism (RoPE) score calculator [20].^a^

Characteristics	Points
No history of hypertension	1
No history of diabetes	1
No history of stroke or transient ischaemic attack	1
Non‐smoker	1
Cortical infarct on imaging	1
Age, years	
18–29	5
30–39	4
40–49	3
50–59	2
60–69	1
≥ 70	0
Total RoPE score = sum of individual points	

^a^ The RoPE score assesses the probability that a patent foramen ovale (PFO) discovered in the setting of an otherwise cryptogenic stroke was pathogenically related to the stroke rather than an incidental finding. The RoPE score ranges from 0 to 10, with scores of 0–3 indicating a negligible likelihood that the stroke is attributable to the PFO and a score of 10 indicating an approximately 90% probability that the stroke is attributable to the PFO.

**TABLE 4:** The Patent Foramen Ovale‐Associated Stroke Causal Likelihood (PASCAL) classification system [20].^a^

PASCAL classification system^a^		
High RoPE score (≥ 7)	High‐risk PFO feature (LS and/or ASA)^b^	PFO‐related stroke
Absent	Absent	Unlikely
Absent	Present	Possible
Present	Absent	Possible
Present	Present	Probable

Abbreviations: ASA, atrial septal aneurysm; LS, large shunt; PFO, patent foramen ovale; RoPE, Risk of Paradoxical Embolism.

^a^ The PASCAL classification system combines the RoPE score with the presence or absence of high‐risk PFO features to determine the likelihood that the PFO was causally related to the index stroke.

^b^ ASA is defined as ≥ 10 mm of excursion from mid‐line. Large shunt size was defined as > 20 bubbles in the left atrium on transesophageal echocardiogram.

The PASCAL classification system addresses the limitation of the RoPE score by integrating it with the presence or absence of high‐risk PFO features (i.e., large shunt size and atrial septal aneurysm) to determine the likelihood that the PFO was causally related to the index stroke [10, 20]. Patients classified as ‘unlikely’, ‘possible’ and ‘probable’ using the PASCAL Classification System had hazard ratios of 1.14 (95% CI, 0.53–2.46), 0.38 (95% CI, 0.22–0.65) and 0.10 (95% CI, 0.03–0.35), respectively (*p* for interaction = 0.003) [20]. The 2‐year absolute risk reduction was −0.7% (95% CI, −4.0% to 2.6%), 2.1% (95% CI, 0.6%–3.6%) and 2.1% (95% CI, 0.9%–3.4%) in the ‘unlikely’, ‘possible’ and ‘probable’ PASCAL categories, respectively [20]. Thus, in line with Kent and colleagues, who state that the ‘application of [the PASCAL] classification system has the potential to guide individualized decision‐making’ [20], the consensus endorses the use of the PASCAL classification system over the RoPE score alone for treatment decisions (*Recommendation 8*).

There is agreement between right‐to‐left shunting detection and grading with TOE and TCD, although there are important examples of substantial right‐to‐left shunting seen only with TCD [40]. Integration of TCD grading results into decision‐making recognises the prognostic value of shunt severity and spontaneous shunting. High‐grade shunts (Spencer grade ≥ 3) and spontaneous right‐to‐left shunting predict increased recurrence risk and may identify patients most likely to benefit from closure [41]. The consensus recognised that TCD grading provides both diagnostic and prognostic information, making it valuable not only as a screening tool but also for risk stratification (*Recommendation 9*).

There is clinical evidence that PFO closure in patients aged < 60 years with cryptogenic stroke and high‐risk anatomical features, such as a large right‐to‐left shunt or associated atrial septal aneurysm, substantially reduces recurrent ischaemic stroke compared with medical therapy alone [17, 19, 42, 43]. For instance, in the CLOSE trial [17], no recurrent strokes occurred among patients treated with PFO closure plus antiplatelet therapy, versus 14 events in those receiving antiplatelets alone (hazard ratio, 0.03; 95% CI, 0–0.26; *p* < 0.001). The DEFENCE‐PFO study [19] similarly found that closure in patients with high‐risk PFO morphology reduced the composite endpoint of stroke, vascular death and major bleeding to 0% versus 12.9% with medical therapy (*p* = 0.013). Meta‐analytic evidence encompassing 6961 patients confirmed a significant decrease in recurrent stroke (odds ratio, 0.39; 95% CI, 0.24–0.63) with closure, particularly among individuals with large shunts or atrial septal aneurysm, albeit with increased atrial fibrillation risk (odds ratio, 5.74; 95% CI, 3.08–10.70) [42]. Further magnetic resonance imaging‐based analysis from the REDUCE trial [43] objectively demonstrated that PFO closure halved the incidence of new brain infarctions (4.7% vs. 10.7%; relative risk, 0.44; *p* = 0.02), largely due to fewer clinical strokes. Collectively, these data establish that for patients with PFO‐associated stroke and high‐risk features predictive of paradoxical embolism, device closure provides superior secondary stroke prevention compared with antithrombotic therapy alone (*Recommendation 10*).

The mandatory requirement for multidisciplinary evaluation reflects international best practice and addresses the complexity of PFO management decisions. Heart–brain teams facilitate a comprehensive assessment of stroke mechanism likelihood, optimal patient selection and shared decision‐making, while avoiding inappropriate interventions. Australian and New Zealand implementation of heart–brain teams requires structured collaboration between neurology and cardiology services, with regular multidisciplinary meetings, standardised referral pathways and shared care protocols (*Recommendation 11*).

The consensus acknowledges that concurrent thrombophilia, deep vein thrombosis or pulmonary embolism may influence treatment decisions, particularly regarding the duration of anticoagulation and the timing of closure. These factors are not currently captured in risk stratification tools, but they may identify patients at a higher risk of recurrence who warrant consideration for intervention. The group recognised that patients with provoked venous thromboembolism may have limited‐duration anticoagulation, potentially leaving them at increased risk if anticoagulation is discontinued (*Recommendation 12*).

### 4.5 Areas of Uncertainty

Several areas of clinical uncertainty were identified during the consensus process, representing questions where evidence is limited and clinical practice varies. These areas require individual patient assessment and may benefit from future research.

For instance, the question of whether patients with known PFO should undergo closure before procedures associated with paradoxical embolism risk, such as sclerotherapy of varicose veins or liposuction, remains unresolved [44]. Although these procedures carry a theoretical embolic risk from the introduction of air or fat emboli, the actual clinical risk in patients with PFO remains unclear.

Next, the relationship between PFO and migraine with aura is well‐established epidemiologically, with PFO found in 46.3%–88% of patients with migraine with aura [45]. However, the role of PFO closure in migraine management remains controversial, with conflicting trial results and no clear consensus on patient selection criteria for this indication.

The presence of PFO in recreational divers raises questions about decompression sickness risk and the need for screening or closure. Despite evidence for increased decompression illness risk in divers with large PFOs [46], routine screening and prophylactic closure recommendations remain unclear.

Finally, patients with both PFO and inherited or acquired thrombophilia present complex management challenges. Questions include optimal anticoagulation duration, timing of PFO closure relative to thrombophilia treatment and whether thrombophilia influences closure benefits. The interplay between these conditions and stroke recurrence risk requires further investigation.

These areas of uncertainty highlight the need for continued research and the importance of individualised patient assessment in complex cases. Clinicians managing such patients should consider multidisciplinary consultation and may benefit from involvement in relevant research initiatives or registry studies.

## 5 Limitations

The recommendations are based on current evidence and clinical experience (primarily from neurologists and one interventional cardiologist) and should be interpreted accordingly. In addition, the feasibility of implementing these recommendations will vary across healthcare settings, particularly regarding the availability of TCD and multidisciplinary team resources.

Future research priorities include region‐specific outcome data for PFO closure, health economic evaluations and implementation studies for recommended care pathways. Long‐term registry data could further refine patient selection criteria and validate proposed approaches.

## 6 Conclusion

This Australian and New Zealand consensus statement provides evidence‐based guidance for PFO‐associated stroke management, emphasising appropriate patient selection, optimal diagnostic approaches and structured treatment decision‐making. Key recommendations include universal screening for patients with cryptogenic stroke who are aged ≤ 60 years, TCD as the preferred first‐line screening method, PASCAL‐based treatment decisions and mandatory multidisciplinary evaluation.

Implementing these recommendations requires enhanced TCD service provision, structured heart–brain team development for multidisciplinary consensus decision‐making and standardised care pathways. This approach has the potential to improve patient outcomes through targeted interventions in suitable candidates, while avoiding unnecessary procedures in those unlikely to benefit.

The panel recognises that PFO management will continue to evolve as new evidence emerges and technological advances unfold. Regular review and updates of these recommendations will ensure that they remain relevant to clinical practice and incorporate new evidence as it becomes available.

These recommendations represent a collaborative effort to standardise and optimise PFO care across Australia and New Zealand, promoting evidence‐based practice and equitable access to appropriate interventions. Their successful implementation requires ongoing commitment from stroke and cardiology communities to multidisciplinary care and continuous quality improvement.

## Author Contributions

**Brian R. Chambers:** conceptualisation, methodology, analysis, writing (original draft), writing (review and editing) of the manuscript. **Lauren M. Sanders:** conceptualisation, methodology, analysis, writing (original draft), writing (review and editing) of the manuscript. **Amanda Gilligan:** conceptualisation, methodology, analysis, writing (original draft), writing (review and editing) of the manuscript. **Carlos Garcia‐Esperon:** conceptualisation, methodology, analysis, writing (original draft), writing (review and editing) of the manuscript. **Jan Ho:** conceptualisation, methodology, analysis, writing (original draft), writing (review and editing) of the manuscript. **John Fink:** conceptualisation, methodology, analysis, writing (original draft), writing (review and editing) of the manuscript. **Matias Yudi:** conceptualisation, methodology, analysis, writing (original draft), writing (review and editing) of the manuscript. **Matthew Lee‐Archer:** conceptualisation, methodology, analysis, writing (original draft), writing (review and editing) of the manuscript. **Vimal Stanislaus:** conceptualisation, methodology, analysis, writing (original draft), writing (review and editing) of the manuscript. **Andrew A. Wong:** conceptualisation, methodology, analysis, writing (original draft), writing (review and editing) of the manuscript.

## Funding

London Agency was engaged to facilitate the meeting and provide medical writing support, which was funded by Abbott Australasia, in accordance with Good Publication Practice guidelines (http://www.ismpp.org/gpp‐2022). However, Abbott Australasia did not participate in the meeting or have any role in determining the topics for inclusion in the consensus statement nor in the presentation of the recommendations. None of the participants received an honorarium, or travel and accommodation.

## Disclosure

Not commissioned; externally peer reviewed.

## Conflicts of Interest

The authors declare no conflicts of interest.

## Supporting information


**Data S1:** mja270199‐sup‐0001‐supinfo.pdf.

## Data Availability

This article includes no original data.
