# Supplementary material for: Diagnosis and Management of Patent Foramen Ovale for Stroke Prevention: An Australian and New Zealand Consensus Statement Developed by a Modified Nominal Group Approach
Source: Med J Aust. 2026 May 13;224:e70199. doi: 10.5694/mja2.70199 (PMC13172030; doi:10.5694/mja2.70199)
Supplement: Supplementary file 1 — Data S1: mja270199‐sup‐0001‐supinfo.pdf. [file MJA2-224-0-s001.pdf]

The Medical Journal of Australia

Supporting Information

Supplementary material

This appendix was part of the submitted manuscript and has been peer reviewed.
It is posted as supplied by the authors.

Appendix to: B. Chambers, L. M. Sanders, A. Gilligan, et al. Diagnosis and Management of Patent Foramen Ovale for Stroke Prevention: An Australian and New Zealand Consensus Statement Developed by A Modified Nominal Group Approach. *Med J Aust* 2026; doi: 10.5694/mja2.70199.

Table S1. ACCORD checklist.

ACCORD
Accurate Consensus Reporting Document

Item No.	Section	Checklist Item (*help text*)	No.
T1	**Title**	Identify the article as reporting a consensus exercise and state the consensus methods used in the title.<br>*For example, Delphi or nominal group technique.*	Title page
I1	**Introduction**	Explain why a consensus exercise was chosen over other approaches.	3–4
I2		State the aim of the consensus exercise, including its intended audience and geographical scope (national, regional, global).	4
I3		If the consensus exercise is an update of an existing document, state why an update is needed, and provide the citation for the original document.	N/A
M1	**Methods**<br>Registration	If the study or study protocol was prospectively registered, state the registration platform and provide a link. If the exercise was not registered, this should be stated.<br>*Recommended to include the date of registration.*	4
M2	Selection of SC and/or panellists	Describe the role(s) and areas of expertise or experience of those directing the consensus exercise.<br>*For example, whether the project was led by a chair, co-chairs or a steering committee, and, if so, how they were chosen. List their names if appropriate, and whether there were any subgroups for individual steps in the process.*	4–5
M3		Explain the criteria for panellist inclusion and the rationale for panellist numbers. State who was responsible for panellist selection.	4
M4		Describe the recruitment process (how panellists were invited to participate).<br>*Include communication/advertisement method(s) and locations, numbers of invitations sent, and whether there was centralised oversight of invitations or if panellists were asked/allowed to suggest other members of the panel.*	4
M5		Describe the role of any members of the public, patients or carers in the different steps of the study.	N/A
M6	Preparatory research	Describe how information was obtained prior to generating items or other materials used during the consensus exercise.<br>*This might include a literature review, interviews, surveys, or another process.*	5
M7		Describe any systematic literature search in detail, including the search strategy and dates of search or the citation if published already.<br>*Provide the details suggested by the reporting guideline PRISMA and the related PRISMA-Search extension.*	N/A
M8		Describe how any existing scientific evidence was summarised and if this evidence was provided to the panellists.	5
M9	Assessing consensus	Describe the methods used and steps taken to gather panellist input and reach consensus (for example, Delphi, RAND-UCLA, nominal group technique).	4

		If modifications were made to the method in its original form, provide a detailed explanation of how the method was adjusted and why this was necessary for the purpose of your consensus-based study.	
M10		Describe how each question or statement was presented and the response options. State whether panellists were able to or required to explain their responses, and whether they could propose new items. *Where possible, present the questionnaire or list of statements as supplementary material.*	5
M11		State the objective of each consensus step. *A step could be a consensus meeting, a discussion or interview session, or a Delphi round.*	4–5
M12		State the definition of consensus (for example, number, percentage, or categorical rating, such as 'agree' or 'strongly agree') and explain the rationale for that definition.	5
M13		State whether items that met the prespecified definition of consensus were included in any subsequent voting rounds.	N/A
M14		For each step, describe how responses were collected, and whether responses were collected in a group setting or individually.	5
M15		Describe how responses were processed and/or synthesised. *Include qualitative analyses of free-text responses (for example, thematic, content or cluster analysis) and/or quantitative analytical methods, if used.*	5
M16		Describe any piloting of the study materials and/or survey instruments. *Include how many individuals piloted the study materials, the rationale for the selection of those individuals, any changes made as a result and whether their responses were used in the calculation of the final consensus. If no pilot was conducted, this should be stated.*	5
M17		If applicable, describe how feedback was provided to panellists at the end of each consensus step or meeting. *State whether feedback was quantitative (for example, approval rates per topic/item) and/or qualitative (for example, comments, or lists of approved items), and whether it was anonymised.*	N/A
M18		State whether anonymity was planned in the study design. Explain where and to whom it was applied and what methods were used to guarantee anonymity.	5
M19		State if the steering committee was involved in the decisions made by the consensus panel. *For example, whether the steering committee or those managing consensus also had voting rights.*	N/A
M20	Participation	Describe any incentives used to encourage responses or participation in the consensus process. *For example, were invitations to participate reiterated, or were participants reimbursed for their time.*	Funding statement
M21		Describe any adaptations to make the surveys/meetings more accessible. *For example, the languages in which the surveys/meetings were conducted and whether translations or plain language summaries were available.*	4
R1	Results	State when the consensus exercise was conducted. List the date of initiation and the time taken to complete each consensus step, analysis, and any extensions or delays in the analysis.	5
R2		Explain any deviations from the study protocol, and why these were necessary.	N/A

		For example, addition of panel members during the exercise, number of consensus steps, stopping criteria; report the step(s) in which this occurred.	
R3		For each step, report quantitative (number of panellists, response rate) and qualitative (relevant socio-demographics) data to describe the participating panellists.	Supporting information Table 2
R4		Report the final outcome of the consensus process as qualitative (for example, aggregated themes from comments) and/or quantitative (for example, summary statistics, score means, medians and/or ranges) data.	Supporting Information Table 2
R5		List any items or topics that were modified or removed during the consensus process. Include why and when in the process they were modified or removed.	N/A
D1	Discussion	Discuss the methodological strengths and limitations of the consensus exercise. *Include factors that may have impacted the decisions (for example, response rates, representativeness of the panel, potential for feedback during consensus to bias responses, potential impact of any non-anonymised interactions).*	16–17
D2		Discuss whether the recommendations are consistent with any pre-existing literature and, if not, propose reasons why this process may have arrived at alternative conclusions.	6
O1	Other information	List any endorsing organisations involved and their role.	N/A
O2		State any potential conflicts of interests, including among those directing the consensus study and panellists. Describe how conflicts of interest were managed.	Competing interest statement
O3		State any funding received and the role of the funder. *Specify, for example, any funder involvement in the study concept/design, participation in the steering committee, conducting the consensus process, funding of any medical writing support. This could be disclosed in the methods or in the relevant transparency section of the manuscript. Where a funder did not play a role in the process or influence the decisions reached, this should be specified.*	Funding statement

From: PLoS Med 21(1): e1004326. https://doi.org/10.1371/journal.pmed.1004326 For more information see: https://www.ismpp.org/accord

Table S2. Consensus recommendations for the diagnosis and management of PFO for stroke prevention in Australia and New Zealand.

Number	Consensus recommendation/statement[a]	Level of consensus (N=10)	GRADE[b]
Patient selection for PFO testing			
R1	Patients ≤60 years with cryptogenic stroke should undergo screening for PFO.	100% (10/10)	A1
R2	Patients >60 years with embolic stroke of undetermined source, absence of vascular risk factors, and no atrial fibrillation detected on prolonged monitoring may be considered for PFO screening.	100% (10/10)	B1
Diagnostic investigations			
R3	The historical gold standard for PFO detection is TOE, but this is not the preferred screening investigation for PFO detection.	100% (10/10)	A1
R4	A TCD bubble study should be considered as a first-line screening investigation for PFO detection.	100% (10/10)	B1
R5	Where TCD is not available, TTE with bubble study is an alternative screening investigation, although it has lower sensitivity for PFO detection.	100% (10/10)	B1
R6	In patients with a positive TCD bubble study under consideration for percutaneous PFO closure, the presence of PFO should be confirmed by TOE. TOE will also reveal high-risk features for stroke recurrence, including large PFO and associated atrial septal aneurysm.	100% (10/10)	A1

Treatment decisions			
R7	A high RoPE score alone does not predict benefit from PFO closure and should therefore not be the principal basis of treatment decision-making.	100% (10/10)	B1
R8	The PASCAL classification system combines the RoPE score and high-risk PFO features and should be incorporated into decision-making for PFO closure.	90% (9/10) *(One abstention)*	B1
R9	Spontaneous right-to-left shunt and high-grade right-to-left shunt with Valsalva detected by TCD bubble study are predictors of increased stroke recurrence risk, and should be incorporated into treatment decision-making.	100% (10/10)	B1
R10	Patients with PFO-associated stroke and high-risk features for stroke recurrence should be considered for PFO closure.	90% (9/10) *(One abstention)*	B1
R11	PFO closure decisions should be made by a multidisciplinary heart-brain team that includes a stroke physician and a structural heart cardiologist.	100% (10/10)	GPP
R12	In patients with PFO-associated stroke, other factors, including concurrent deep vein thrombosis and pulmonary embolism and demonstration of thrombophilia, should also be considered in treatment decision-making.	100% (10/10)	GPP

GPP, Good Practice Points; GRADE, Grading of Recommendations Assessment, Development, and Evaluation; PASCAL, Patent Foramen Ovale-Associated Stroke Causal Likelihood; PFO, patent foramen ovale; RoPE, Risk of Paradoxical Embolism; TCD, transcranial Doppler; TOE, transoesophageal echocardiography; TTE, transthoracic echocardiography.

[a]Please see main text for additional context and references to supporting evidence for each of the recommendations.

[b]Recommendations were graded using the GRADE framework. The quality of the supporting evidence was defined as high (A), moderate (B), or low (C) and the strength of the recommendations was classified as strong (1) or conditional (2). Where specific evidence was lacking and recommendations were based on best-practice standard of care, consensus recommendations were designated as GPP. Based on the GRADE framework, high quality evidence (A) was assigned when it was assessed that 'further research is very unlikely to change our confidence in the estimate of effect', moderate (B) when 'further research is likely to have an important impact on our confidence in the estimate of effect and may change the estimate', and low (C) (combining the 'low' and 'very low' categories of the standard GRADE system) when 'further research is very likely to have an important impact on our confidence in the estimate of effect and is likely to change the estimate' or 'any estimate of effect is very uncertain'. Recommendations were assigned as strong (1) when a 'panel is confident that the desirable effects of an intervention outweigh its undesirable effects', and conditional (or weak) (2) when 'the desirable effects probably outweigh the undesirable effects (weak recommendation for an intervention) or undesirable effects probably outweigh the desirable effects (weak recommendation against an intervention) but appreciable uncertainty exists'. The strength of the evidence and recommendations was determined by considering the balance of benefits and harms, the level of certainty, the quality of the supporting evidence, associated costs, resource implications, and patients' personal values and preferences.
